# Outdoor Playground Design Criteria Development for Early Childhood Development: A Delphi Study from the Perspective of Fundamental Movement Skills and Perceptual-Motor Skills

**DOI:** 10.3390/ijerph18084159

**Published:** 2021-04-14

**Authors:** Heejun Jeon, Sunhye Jun

**Affiliations:** 1 Graduate School of Education, Chung-Ang University, Seoul 06974, Korea; xenojun@cau.ac.kr; 2 Department of Physical Education, Chung-Ang University, Seoul 06974, Korea

**Keywords:** outdoor playground, childhood development, fundamental movement skills, perceptual-motor abilities

## Abstract

The purpose of this research is to discover adequate and environmental outdoor playground design criteria based on the collected opinions of experts. Taking the case of South Korea into account, this research focuses on the development of a new outdoor playground design criteria that could offset the limited motor activities and environmental use which has been restricted by the current regulations. In particular, this research approaches the issue from the perspective of children’s fundamental movement skills and the development of their perceptual-motor abilities. The research conducted three rounds of a Delphi survey and held a consensus of experts in child development, child physical education, and playground designs. The research achieved results defining 9 categories and 23 items within the categories of design criteria for outdoor playgrounds. Significantly, the discussions were not limited to children’s holistic development—from motor and physical development to attaining mental, social, and cognitive skills—but also included discussions of children’s dynamic interaction with the surrounding nature and environment, especially in challenge making and risk taking.

## 1. Introduction

Ever since early childhood development has been globally discussed under the pervasion of “education for all”, outdoor play experience in early childhood has been perceived as important for building a solid foundation for one’s lifelong learning and wellbeing [1,2]. This sense of quality, sustainable care, and education for young children lies in its relation to their holistic development: in cognition, physical strength, mental health, social relationships, etc. [3]. Along with issues of family relationships, nutrition, child rights and child care initiatives, the school curriculum, etc., many previous researchers have already drawn attention to the paradox of strengthening learning through play in this period of life [4]. Alongside with interest in the designing programs for effective play, providing a proper learnable environment for these activities to be held should also be thoroughly considered [5]. Akin to the holistic development of a child, designing outdoor play spaces to adjust to nature and enhance childhood perception and motor abilities arose as a primary task for child development [6,7].

A research question that embraces general discussions on outdoor play activities is what the standards are for outdoor playground design suitable for children’s overall development. Recently, with higher concerns yielded from increasing screen time and insufficient exposure to natural and spontaneous outdoor play activity in occupied cities, research on the effect of outdoor play on children’s holistic development has been discussed regarding different dimensions of outdoor play [8], play context and social skill building with peers and parents [9,10], and also the integrity of outdoor playgrounds and nature environments [11,12]. It has been believed that sophisticated designs of outdoor playgrounds elevate not only children’s physical motor skills, but also their cognitive skills, language use, and social ability [13,14]. Additionally, by increasing interpersonal contact, this play space contributes to socialization and provides space for different types of communications to be shared [15]. Especially in terms of an integrated playground for children with disabilities and non-disabled children, the final design should be able to satisfy people of all needs [16,17]. Furthermore, this requires the process and the result to all contribute to the development of social relationships [18], which is an exemplary case of social inclusion. A large and growing body of literature has investigated finding ways to add adventurous elements, successive challenges, accessibility, and links to local society in outdoor playgrounds [19,20,21]. However, much of the research up to now was not able to state a general agreement on the design criteria for outdoor playgrounds from a child’s developmental perspective. Previous traditional playground design criteria are facing challenges to reflect and embrace current issues in early childhood care and education. 

Regarding integrated playgrounds, its advent is closely related to the change of general perspective on social support for children with disabilities [22]. In traditional forms of playgrounds, playground design for children with disabilities was not a matter of importance and was not highly considered in their designs. However, starting a few decades ago, the urgency of upheaving attention of play for children with disabilities began to grow, which led to drawing up policies for greater accessibility to play environments and designs for integrated play programs [23]. Despite devoting efforts to review the existing public play regulations and improve play environments [24], the challenges surrounding children with disabilities in their play activity is still an ongoing issue. 

When it comes to the question of why playgrounds are important for childhood development, it is hard to leave out the discussion of providing educational space for effective learning [5]. Previous studies have given prominence on how designing educational spaces could increase the learner’s interest and performance in learning and also illuminate the learning climate [25,26,27]. This research, especially from the viewpoint that play space for young children could affect their fundamental motor skills in terms of lifelong learning and holistic development, aims to provide one of the first investigations into how the arbitrary traditional outdoor playground designs could be renewed to enhance their sustainability from the perspective of children’s fundamental movement skills and perceptual-motor abilities.

While research in indoor activities for children has tended to focus on the more structured and controlled environment, outdoor play has held significance in that it approaches children’s play activity with less tension and more flexibility in customizing and harmonizing with the natural settings provided—allowing users to engage in more dynamic motor movements [28]. In this regard, through the release of tension from the indoor experience, environmental stimuli, and gross motor movements, outdoor play can bring about the development of children’s physical, social, emotional, and cognitive growth [1,2,13]. Besides this, outdoor play is contemplated to integrate with the learning environment, bridging not only the expansion and support of the indoor experience, but also weighing the priority on fulfilling its imperative function in qualitative parenting [29]. 

With this concern, some leading countries such as the United States, Canada, Australia, etc. have pioneered research in new designs for childhood outdoor playgrounds [30]. However, with fundamental differences in culture, interest, and circumstances, the importance of reforming and redesigning outdoor playgrounds may not be the priority for childhood development in all countries. In this sense, South Korea (Korea) has been a country deficient in taking initiatives for rethinking child outdoor playgrounds—highly recognized for its lack of appropriate outdoor activities compared to its excellence in national curricula (the Nuri Curriculum in Korea) [17,31].

The implications driven from this study on Korea represent relevant considerations for countries that have or may consider stating a nation-based curricular and standards for early childhood education. Although the national curriculum designates physical exercise as one of the five key areas in childhood education, setting the environmental criteria for where outdoor play is held and what it should look like may not have been a prioritization compared to more general interests. Likewise, few researchers have been able to draw on any systematic research into a sustainable outdoor playground design in young children’s motor development perspective—especially from that of Korea where the public education system, including kindergarten education with the national curriculum, takes responsibility for children’s physical, social, and emotional development. Beltzig, as an acknowledged playground designer, has mentioned how Korea has not extensively considered young children’s developmental perspective in modern playgrounds [32]. According to his opinion about Korea’s outdoor playgrounds, most of the open-access playgrounds, educational institution playgrounds, and other outdoor playgrounds of different facilities are cramped and, with relatively poor conditions in their facilities and areas, are not suitable for young children to play from a developmental perspective [32]. This suggests the need for current research in this field to compensate for the shortcomings of traditional playgrounds and to produce outdoor playgrounds designs suitable for physical development in early childhood. 

Prevailing criticism on the traditional outdoor playground designs argues that it is static and boring with fixed elements, and outdated in considering modern children’s perspective on playground use as an important aspect of design [30]. As it has been previously mentioned, it is surprising to see that countries that are leading the settlement and sustainability of childhood care and education by policy and amendments—represented by Korea in this research—have failed to think out of the few general criteria stated in the old systems. Furthermore, these criteria do not take the aspects needed for children’s outdoor activity and development into deeper consideration.

The purpose of this study is to analyze outdoor playgrounds in terms of a child’s perceptual-motor abilities and fundamental motor skills development. In recent studies on childhood educational environment design, the sustainability of the design holds high interest. Particularly, this means that the recent trend in this field perceives to eliminate negative environmental factors through skillful, sensitive, and thoughtful designs [33]. The significance this study holds is that it has taken the approach to collect voices of experts from different related fields on the proper design from the perspective of children’s lifelong motor development. This study provides an important opportunity to advance the understanding of outdoor playgrounds that could impact children’s development factors and suggests a design standard that allows users to naturally enjoy the process of playing and seeking unconscious development.

The paper is organized as follows. Section 2 reviews the theoretical framework of the researcher’s scope of interest in fundamental movement skills and perceptual-motor abilities in early childhood, following how it accounts for play activities and outdoor playgrounds. Furthermore, this section gives an explanation on how the researchers conducted a Delphi study to examine the playground design criteria from the view point of field experts. Next, the results of the study are stated in Section 3, and Section 4 gives an in-depth interpretation to the main findings of the research. 

## 2. Methodology

### 2.1. Scope of Interest

#### 2.1.1. Fundamental Movement Skills and Perceptual-Motor Abilities in Early Childhood

Development refers to numerous changes that occur in human life, including physical, functional, and mental changes. The development of early childhood can be described as a biopsychosocial model that includes inheritance from parents, biological characteristics (represented by child temperament and attachment to mothers), environmental interactions, and social surroundings [34,35]. Particularly, rapid growth in infancy is acknowledged to integrate each aspect of social, emotional, cognitive, linguistic, and physical development and it does not end at a point in childhood, but successively continues to affect generic growth [35].

Motor development in normal childhood has been recognized to have significant implications across human behavior—including physical and emotional stability, motivation and creativity, social adaptation, leisure, and sound personality development [3,36]. Gabbard [37], classifying motor development into “phases” from a holistic biological point of view, argues that motor skills of human beings develop and degenerate in a continuum system of lifelong motor development: referring a person’s prenatal stage as reflexive movement phase (from birth to 6 months old), infancy stage as fundamental movement phase (from 6 months to 2 years old), later-infancy as sport skill phase (from age 6 to 12), adolescence as growth and refinement phase (from age 12 to 18), adulthood as peak performance phase (from age 18 to 30), and older adulthood as regression phase. This explains the linear stage of the development and atrophy of human motor capacity.

Particularly, fundamental movement skills (FMS) are important because they have positive impact on various developmental areas of children [38]. Specifically, FMS plays an important role in social and emotional development such as enhancing self and social awareness, self-regulation, leadership, responsible decision-making, self-concept, and peer competence [39], as well as cognitive development represented by language and learning competence [40,41]. Thus, the acquisition and refinement of FMS in early childhood plays a positive role in social, emotional, and cognitive development as well as physical development [38]. 

According to the motor development lifespan, children from the age of 2 to 7 are noticed to be the stage of developing FMS [36]. These skills include three important aspects: stability, locomotion, and manipulation, required for efficient body movement in their future [42]. This framework of the three aspects is proposed in learning “to” move and learning “through” movement in school contexts [42]. The three developmental features of FMS were proposed by Jeon in Table 1 [43].

Creating a functional and practical link between kinder-kinetics and perceptional-motor abilities is a task that tags along in early childhood development [44,45,46]. Perception refers to a series of processes that accept a given stimulus from an external environment or from one’s inner self and transmit it to the brain, which can also be seen as a process of integrating, interpreting, and re-controlling various movements [36,47]. Logically, the two concepts—perception and motor—can be set aside independently. Yet, motor development based on perceptual-motor abilities, which integrates the two concepts, holds importance in providing children basic learning concepts, integrating neural information, proceeding motor control, etc. [44,48]. A great deal of previous research also started from the fact that youth with assorted experiences of motor and perceptual learning have a greater opportunity of perceptual-motor association—amplifying their ability to cope with challenging physical situations flexibly. 

According to Gallahue [42], perceptual-motor abilities are specified in four types: body awareness (e.g., understanding location, shape, and muscle tension/relaxation of different body parts), spatial awareness (e.g., building self-space and respecting other’s personal space, understanding concepts of height, length, area, etc.), directional awareness (e.g., understanding different directions, learning how to change direction), and temporal awareness (e.g., understanding concepts of speed, days/hours, historic time, etc.) (see Table 2). Especially in early childhood, hand-eye coordination is one of the most important perceptual motor abilities needed for lifelong motor development. This is the ability to coordinate accurate and efficient hand-oriented movements based on the information collected from the vision system [49,50]. This means that all different kinds of movements—from basic daily-life movements (such as using tools to cook, wash, eat, etc.) to achieving particular goals (such as studying, playing music, playing on a sports team, etc.)—require perceptual-motor abilities development. Research suggests that it is timely for young children, when a leaping change occurs in their visual perception, to upheave their neuro-physiological development through the occupation of proper and sufficient movement activities [49,50]. 

#### 2.1.2. Value of Play, Outdoor Play, and Playgrounds

Researchers in child development claim that play is an important part of perceptual, physical, social, and emotional development for children and that they can learn about themselves and their world in the process [1,2,4]. Playing helps children to learn how to control their bodies through physical movement and develop motor skills. The movements obtained during the play not only promote the development of the child’s body growth, but also teach them how to express using their body [7,8,10]. In the discussion about the value and importance of play in children’s development, children also develop cognitive skills (e.g., spatial ability, logical-mathematical skills, and verbal and visuospatial memory skills) through body movements in playing [52]. In this activity, they go through a spontaneous cognitive learning process. While covering different subjects to play and using particular objects in their play, children are open to observing and learning physical phenomena of shape, color, size, and texture of them. 

Particularly in regard to outdoor play, this helps children with systemic coordination and balanced development, hand-eye coordination, and skeletal-muscle development [11,12,19,42]. Outdoor space can give children more mental and physical freedom in less organized spaces for playing activities. This also vitalizes the momentum for children to spontaneously select to choose the activities they prefer and to take the initiative in more physical and complicated activities—with some even being challenging and risky [19,21]. Likewise, outdoor play itself represents a child’s regular life and learning process; as well as a way of expressing oneself and engaging the most stimulating factor that affects children’s development of fundamental motor skills and perceptual-motor abilities in advance. 

However, traditional playgrounds are often thought of as a place of limitation where only physical, active movements happen. This space functions in a complex where a child’s holistic development is promoted. This cannot be ignored regarding the growth of a child. The primary function of an outdoor playground is to promote play, but it also functions to promote the learning and development of children [6,8,10]. Outdoor plays provide a variety of problem-solving opportunities to activate convergent and divergent thinking, and teach one how to wisely cope with situations where creative thinking is requested [53]. Furthermore, young children’s outdoor plays affect their spatial awareness, complex mental mapping of the area, and sense [4,29]. Thus, future-oriented playgrounds should be a place where children can freely engage in peculiar physical activities, providing extraordinary and rich sensory experiences, along with creating multi-relationships. Furthermore, allowing risky play in outdoor playgrounds is one of the essential factors for the development of children. This generates fresh insight into outdoor playground design, which tends to limit risky action and movements from happening to protect children from danger [21,54].

A distinct feature of Korea’s childhood play in early childhood care and education is that specific standards for childhood outdoor playgrounds are practiced by law. This has led researchers to take the initiative of relating childhood development features with playground environment studies [55]. Such approaches to playground designs, however, have failed to address the specific development of childhood motor skills as a priority. On the other hand, referring to the implications of previous research, new expectations in this field also include bringing children more freedom and challenges by eliminating the regulations practiced under the principle of children safety and tearing down the fences in between the nature settings and artificial playground [32]. 

### 2.2. Research Sample and Data Collection

To develop an outdoor playground design standard in terms of perceptual-motor abilities and fundamental motor skills, two panel groups with expertise and knowledge were formed. With a total of 40 panels in the Delphi study, 22 panels of infant and child experts and 18 panels of playground experts participated. 

Prior to the official Delphi study, this research conducted a pilot survey based on the results of literature reviews and in-depth interviews (see Appendix A). The researcher collected survey results from five experts between March and April 2018 via e-mail and revised and restructured the survey questionnaires for the round one survey. Between April and October 2018, the first round of open-ended survey answers and opinions of the panels were collected on developing design standards for outdoor playgrounds. These responses were classified through content analysis. 

Based on the questions and responses from the first round, the survey for the second round was reconstructed with five-point scale response questions on the conformity and orientation of outdoor playground design criteria. The questions for the third round went through a refinement process based on the questions and responses from the second round, and re-verified the previous responses on their conformity and orientation. As a result, 18 of the 40 total panels were selected to be valid responses. Specific information on the profile of the panels is clarified in Table 3.

### 2.3. Delphi Study

A Delphi study is a method of formalizing the “intuitive judgment” of experts through the process of repeated surveys [56]. It is a method of asking a group of experts (each of it called a panel) through a survey. Each of the sequential surveys is referred to as a “round.” The questionnaires in this process not only collect survey results, but also solicit advantageous information, such as consensus and pros and cons on the topic from experts, and make the exchange of their opinions and ideas possible in solidly designed research [56]. 

**Figure 1:**
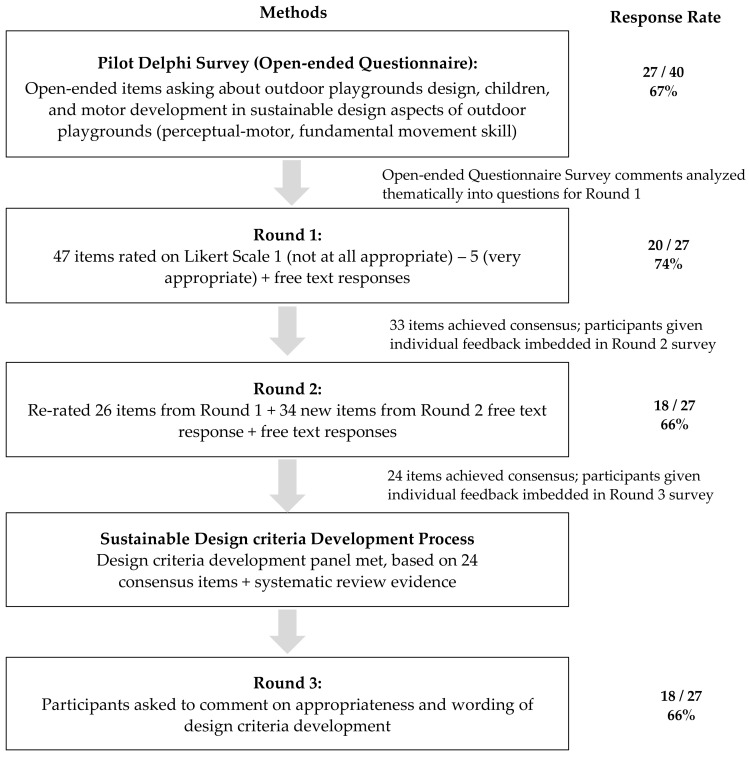
Steps of the Delphi study method.

The goal of a Delphi research is to clarify and materialize the uncertainty of the future by repeatedly asking the intuition of the experts on the future. The Delphi method is an alternative survey method for the collective consultation method that elicits consensus on the questionnaires from the subjects. It repeatedly sends out surveys to planned anonymity and takes the feedback on previous results into consideration. In this research, the Delphi method was proceeded in the following order, as shown in Figure 1. First, it selected a panel of experts in the relevant field. After two rounds of questionnaire feedback based on the research purpose, the facilitator achieved the final stage of building consensus on the research agenda.

### 2.4. Data Analysis 

Through the peer-to-peer review and member review based on the results of the first survey, questions were added, integrated, renamed, and removed for the following stages of the Delphi study. To verify the suitability and validity of the detailed categories and components in the second and third round, which mostly constituted closed-ended questions, the researcher went through a descriptive statistical analysis (SPSS 21.0 ver.). Specifically, frequency analysis, intraclass correlation coefficient (ICC) analysis, and Kendall’s Coefficient of Concordance W were conducted. The ICC validation is a correlation coefficient used to indicate reliability when a measurement is quantitative, which represents the correlation between the measurement tools, or evaluators in a sample. The higher ICC is, the higher the reliability is—typically, values over 0.8 can be interpreted to have strong validity, and values over 0.6 to have relatively strong validity [57]. This research also used Kendall’s Coefficient of Concordance W and content validity ratio (CVR) to secure consensus among the Delphi experts. When the results showed statistical significance, the panels’ opinions were interpreted as mutually agreeable. 

Last of all, the validity of the Delphi study was assessed using CVR based on the theory by Lawshe [58]. The level of consensus of the expert group was obtained through the equation shown below.

(1) $$CVR=\frac{Ne-\frac{N}{2}}{\frac{N}{2}}$$

where *Ne* is the number of experts who responded that an item is important (with a Likert scale 4 or 5) and *N* is the number of the whole response [58]. The CVR value was determined by the total number of experts participating in the Delphi survey. With a total of 18 panels involved, this research defined that the items had content validity when the CVR value was higher than 0.45, with its p-value lower than 0.05 [58]. Furthermore, referring to Kline [59], we considered that regular distribution could be found when the absolute value of the skewness did not pass 3.0 and we assumed a normal distribution in analyzing the final results.

## 3. Results

The sequential sections that follow are the results found from the three rounds of Delphi survey. They refer to the collected consensus on design criteria adequate for outdoor playgrounds from the perspective of children’s perceptual-motor abilities and FMS. In this research design criteria refers to conceptual standard of the fundamental goals that a project must achieve throughout the comprehensive process of designing and problem-solving. That being said, the design criteria for outdoor playgrounds attained from this research finding contain the basic items of consideration necessary for playground designers. 

### 3.1. Round 1

In the round 1 survey, panels were given an open-end questionnaire survey of four questions covering what motor developmental factors (perceptual-motor abilities and FMS) outdoor playgrounds should cover, what design criteria are needed to perceive motor development, what suggestions exist for developing the design criteria, and what limitations exist in the prevailing playground designs.

The respondents showed similarities in their answers on the limitations that traditional playground designs have on child perceptual-motor abilities development and FSM. The three groups of expertise expressed different approaches and evidence for the need of new design criteria. In case of childhood education experts, one of responses shed light on the existence of different types of play (comprehensive play, physical activity play, and autonomous play). Furthermore, they pointed out that playgrounds should offer children with more opportunities to engage in adventures and a wider area for those motor skills to be practiced safely. More focused on the motor development in childhood, experts in childhood physical education provided more practical ideas to induce motor movements. Moreover, experts implied that making the playground to coexist with the natural environment is a new task in terms of enhancing motor development. Specific results from round 1 of the Delphi study are stated in Table 4.

According to the first Delphi survey, the design criteria for playground design were set to be categorized into nine categories: effectiveness (five criteria), innovation (three criteria), diversity (four criteria), interest (four criteria), efficiency (five criteria), complexity (three criteria), stability (two criteria), relationship (three criteria), and connectivity (four criteria)—a total of 33 items out of 47 were proposed to the panels. This result was used in the following round of Delphi.

### 3.2. Round 2

On the basis of the results from the first round, the round 2 survey questionnaires were revised and adjusted through a verification process of peer-to-peer review and the Advisory Committee validity check. The researchers had multiple meetings with the Advisory Committee for validity and reliability tests. The Advisory Committee was constituted with one expert in psychology, one expert in sociology, three teachers/instructors specialized in early childhood education, and three teachers/instructors specialized in early childhood physical education. The Advisory Committee participated in the whole process of the research from revising the drafted questionaries that the researchers had driven out from the answers in the open-ended question survey. The role of the Advisory Committee was to discuss and determine the survey questions derived from experts’ feedback, analyze the results of the response, and collect and compile opinions throughout the correction of questionaries to raise the validity. The answers from round 2 were analyzed to verify the validity of the items in the previous category. The results to the second round are reported in Table 5. Here, the shaded cells show the items in which the CVR was lower than 0.50, based on the number of respondents. All of the nine categories were found to be valid, but there were some changes to the specific items in each of the categories. The items drawn from the results of round 2 with the conformity of a CVR value higher than 0.50 are stated in Appendix B.

### 3.3. Round 3

Most of the items derived from the Delphi round 2 were reverified as appropriate initiatives. Exceptionally, one item from the relationship which was found to be somewhat less appropriate was rejected in the third round.

The results of Delphi round 3, systematically refined with the results from the second Delphi survey, reconfigured the former criteria to a total of 23 items within the 9 categories (Table 6).

Specifically looking into the results of the third round, out of the nine categories, the consensus of experts on the diversity design criteria was found to be strong. In addition, the panels expressed their support on the point that young children should be capable of proceeding complex functions from the perspective of perceptual-motor abilities and FMS; also, they supported that its environment should be constructed of a mix of different materials. so that children could have a wide sense of experience. Regarding the design from the view of play activity, the necessity of the diversity criteria is notable from the experts’ accordance on the item that outdoor playgrounds should be designed for users to be capable of the most diverse experience. The following are the core contexts that were emphasized in the finding:

1. The effective design criterion mainly refers to the degree of goal achievement. Here, the development of potential mobility and application of the design from which children could reach higher achievements from challenging their goals in the space was emphasized;
2. The innovation design criterion means changing the old to the new. The application of user-centered designs, breaking away from the former traditional playgrounds, was emphasized in this criterion;
3. The diversity design criterion refers to various characteristics such as shape and style. It emphasized whether or not the design could encourage young children to attempt inaccessible and unpredictable experiences;
4. The interest design criterion consists of factors related to the enjoyment users can feel, or excitement. Storytelling is an important aspect in the related activities—far from that of fragmented activities;
5. The efficiency design criterion refers to what can be achieved with minimal effort. Here, the interaction of young children and the space and a design without de-developmental obstructions is important;
6. The complexity design criterion refers to the combination of two or more different functions—such as physical strength, perception, and exercise development. The design ought to allow young children to achieve two or more developments simultaneously through activity in the space;
7. The stability design criterion seeks for the playground to remain unchanged and constant, emphasizing whether an individual is capable of stable activity on their own and whether a fixed and unchanging design of the facility material is guaranteed;
8. In the relationship design criterion, two or several targets are connected. A playground should be designed for youth to interconnect between individuals and with the surrounding environment;
9. The connectivity design criterion refers to the interaction with particular events or phenomena. Through activities in the area, physical development can occur from naturally and unconsciously activated movements of body-part coordination and fundamental movement skills.

## 4. Discussion

On the basis of the founding that the physical environment of outdoor playgrounds affects child development, the purpose of this research was to establish design criteria for sustainable outdoor playgrounds by taking the view of perceptual-motor abilities and fundamental movement skills into account. According to Chung [21], prevailing traditional outdoor playgrounds—far from what users, especially, parents, would expect—have a negative impact on the potential development of young children due to the limited play environment and its composition of typical back-number elements. Coming across this issue in Korea, the research also states that it has become more crucial in recent years as younger children have started to spend most of their time in daycare centers after the Free-Childcare System settled. In addition, the trend found in the current generation of increased screen time and less motor activities is hard to ignore [10]. The environment these children are exposed to could improve, or rather disturb, their physical, language, and social skills as well as creativity.

Although Korea is recognized for a well-formed and pioneering national curriculum for early childhood education, it is one of the countries that has outdated and limited types of designs for outdoor playgrounds; this indicates that most of the play environments offered are centered on the constitution of elements, which takes merely a narrow part of the discussions on possible outdoor playground designs into consideration. With rising expectations on outdoor playground design standards, research on outdoor playgrounds adequate for holistic youth development is of paramount importance. On the one hand, unlike the past, when disability-related policies only focused on treatment and rehabilitation, interest in suitable outdoor playgrounds for both disabled and non-disabled students to enjoy has started to rise. This is why emerging designs for outdoor playgrounds tend to put more effort in bring about “social integration” [16]. For this reason, the originality of this study is that it explores a sustainable design standard for young children’s fundamental movement skills and perceptual-motor abilities development from their play activity at outdoor playgrounds. The results of research are discussed below.

First, throughout the Delphi survey, the majority of the panels settled in agreement that high-intensity, gross motor activity is appropriate for outdoor playground activity, rather than static activity. Although they also showed concern on the limitation of the current outdated designs, they expressed their belief that, if applicable environments are established, manipulative movement skills can also follow in a well-designed outdoor playground when children’s perceptual-motor abilities and FMS development is regarded. In the open-end responses, many asserted that encouraging active muscle movements in outdoor playgrounds is adequate for early childhood development as opposed to static activities. Especially for manipulation skills, many from the panel predicted that there would be difficulties in outdoor playgrounds designs to technically embrace the needs for developing these skills. However, they still held on to the idea that manipulation movement skills should be implicated in outdoor playground designs in the end. One example we found this in is “the Imagination Playground” in Bruling Slip, New York [60]. This playground is an open field with toys and tools that catch children’s curiosity and could help them advance their manipulation movement skill thoroughly. This well explains the possibility of applying related criteria as a general aspect for outdoor playgrounds in the near future.

Second, out of the four perceptual-motor abilities, the panels showed a strong consensus on the need of body awareness and spatial awareness. This is the result of accepting the panel’s thought that recognizing and understanding one’s body and their activity space is the basis of making profit out of the time spent in the playground. Unfortunately, the panels answered that the speed concept in temporal awareness may have a risk of dragging some harmful factors into the children’s activity, and these factors showed the lowest importance. Thus, despite the need for risk taking in children’s activity in outdoor playgrounds, at least in relation to time perception in the concept of speed, designers may need to be cautious when they apply it in the design. The younger the users are, the more the concept should permeate naturally into their activity in the playground.

Third, collective responses of the perception that panels have on the current playground designs argued that they have functionally reached their limit, emphasizing the importance of creating an outdoor playground environment that triggers continuous interaction (e.g., user and the facility, user and the environment, interaction between users, etc.). Their idea is supported by Solomon [30] who has claimed that children should be applicable to constant and dynamic free-play, have the opportunity to face positive risks and challenges of unpredictable situations, and engage in intercooperation during their time in outdoor playgrounds In this regard, this research hold a significance on the fact that it has critically approached the formal design standards to reform them to a sustainable playground design from the perspective of children’s perceptual-motor abilities and FMS development. 

Given that the panels who participated in this research are experts from both the fields of young children’s physical activity and their play environment, it can be concluded that the findings hold strong solidity in reflecting the educational and social goals of outdoor playground construction. Moreover, with a clear-cut consensus derived from those practitioners and expertise closely working in the field, the sustainable design criteria developed in this work raise a question on the typical and synchronized designs of playgrounds often seen today [32]. 

## 5. Conclusions

Although their motor skill levels were quite low, the children generally held positive perceptions of their physical competence. These positive perceptions provide a window of opportunity for fostering skillfulness. The modest relationships between perceptions of competence and motor skill proficiency suggest that the children are beginning to make self-judgments at a young age. Accordingly, opportunities for children to become and feel physically competent need to occur early in their school or preschool life.

This research proposed a design basis for motor-developmental outdoor playgrounds based on relevant experts of childhood development and their play activity with differentiating experience and expertise. In particular, the process of collecting and adjusting the arguments of different panels from different fields was of substance. Therefore, a further study that could hear from experts in various fields such as parenting, design, and landscaping, as well as practitioners from park management, is suggested. 

The reader should bear in mind that the subject of this research is limited to outdoor playgrounds generally found in children parks or residential complexes. This provides abundant room for further research to discover different forms of playgrounds—such as those of childcare institutes—from the perspective of perceptual-motor abilities and FMS development to stress their importance. Furthermore, because the primary goal of this research was to provide a basic guide to the youth outdoor playground, it was not the task of this research to specifically design a playground from the perspective of its interest. To develop a full picture of the playground design that this research suggested, additional studies are needed to map out and do the drawings for the practical foundation of the outdoor playground from the perspective of young children’s perceptual-motor abilities and FMS development.

As stated earlier, the implications driven from the case of Korea have significance in that this research could provide practitioners with information to relate the designs of the national curriculum and environment with considerations of physical development in the early years. However, readers may need to have a sense of cautiousness in applying the criteria in a different context, culture, region, etc. The result from this research could provide the basis for playground design in the view of perceptual-motor ability and FMS development, but subsequent research could also examine new forms of outdoor playgrounds by adding different aspects of childhood development and environmental and social change. 

The next step to utilizing the results of this study is to develop practical guidelines for outdoor playground designs that pursue social sustainability. If this succeeds, it could contribute to the composition of outdoor playgrounds in terms of perceptual-motor abilities and FMS development; functioning as a catalyst for achieving higher levels of children’s physical and holistic development. In addition, it can affect the fundamental development of outdoor playgrounds, especially in countries where motor-developmental design standards have not yet been established.

## Figures and Tables

**Table 1:** Components of Fundamental Movement Skills.

Stability Movement		Locomotion		Manipulation	
Axial Movement	Static and Dynamic	Basic	Combination	Propulsive	Absolvent
Bending Stretching Twisting Turning Swing	Upright balance Inversed balance Rolling Starting Stopping Dodging	Walking Running Leaping Hopping Jumping	Climbing Galloping Sliding Skipping	Ball rolling Throwing Striking Kicking Bouncing Punting Volleying	Catching Trapping

Table is adapted from developmental features of FMS proposed by Jeon [43].

**Table 2:** Factors Associated with Perceptual-Motor Components.

Factor	Example
Body awareness	• Knowledge of body parts • Knowledge of what body parts can do • Knowledge of how to make body parts move efficiently
Spatial awareness	• Subjective localization • Objective localization • Self-space • General space
Directional awareness	• Laterality • Directionality
Temporal awareness	• Synchronization • Sequence • Rhythm

Table adapted from explanation of perceptual-motor components proposed by Goodway, Ozmun, and Gallahue [51].

**Table 3:** Summary of Delphi Study Panels’ Information.

Sort of Panels	Selection Criteria	Participants
Infant and Early Childhood	PhD in infant and early childhood research An expert in infant and early childhood education and has experience in instructing classes in the field for 5 years or more	3 professors and 2 principals in early childhood education institutions
	PhD in early childhood physical education research An expert in early childhood physical education and has experience in instructing classes in the field for 3 years or more	7 professors
Play Infrastructure and Play Environment	Has experience in instructing classes in play environment Has been in work related to play infrastructure for 10 years or more	6 professors and instructors and 2 researchers

**Table 4:** Representative Response on Outdoor Playground Design Criteria Initiative.

Expert Panels	Responses
Childhood Education	Various types of play facilities should be created to reflect the characteristics of children’s development so that they can vent their desire for play. An aesthetic and safe playground needs to be created so that fundamental motor skills and perceptual-motor abilities can be expressed. A facility space and area where comprehensive play (where children with diverse body features and senses can integrate), physical activity play (where children can be free of limits on various forces and speeds), and autonomous play (which could be voluntary used to recreate the play environment) are possible should be produced; the play facility and factors need to be arranged for children to challenge themselves and engage in adventures.
Childhood Physical Education	Motor development in early childhood has a significant impact on the motor performance ability in adulthood. Early childhood is also a critical period for ensuring safety and attaining healthy and quality of life. Therefore, it is necessary to secure a creative playground for various motor development and balanced physical development of young children. In order for users to sprint or run, at least 18 m of empty and safe space without obstacles is required. Furthermore, the facilities constructed must be nature-friendly and real-life-oriented so that users can walk, rip, hop, jump, gallop, slide, skip, etc. Manipulative movement skills: actions such as striking, kicking, and volleying can risk the injury of users and the arrangements of the elements of the playground could hinder their activities. Therefore, it is necessary to secure separate spaces for these activities.
Play Facility and Playgrounds	Make it a playground that coexists with nature. The elements, surrounding nature scenery, and the ground can all be considered as factors of a playground. In many cases, under the protection and restrictions of landscape management, the natural surroundings are often beyond reach of being re-designed as a play element. A playground with storytelling should be configured. The present design system, which is determined mostly by which design company is selected, leaves out customizable factors in different regions, which cannot create interest and motivation for users. Similar combination-based playgrounds reduce the playground industry—upheaving the dependency of developed countries—and could become an obstacle in Korean playground industry.

**Table 5:** Conformity and Validity Results on Outdoor Playground Design Criteria Development Proposal.

Sort	Items	M	SD	Skewness	CVR	ICC	Kendal’s W
Effectiveness	EFT1	4.25	0.856	−0.546	0.50	0.938 ***	0.000 ***
	EFT2	4.25	0.856	−0.546	0.50		
	EFT3	4.19	0.834	−0.391	0.50		
	EFT4	4.88	0.342	−2.509	1.0		
	EFT5	4.69	0.704	−2.082	0.750		
Innovation	INN1	4.75	0.447	−1.278	1.0		
	INN2	4.06	0.998	−0.598	0.375		
	INN3	4.50	0.632	−0.904	0.875		
Diversity	DV1	4.25	0.775	−0.492	0.625	0.938 ***	0.000 ***
	DV2	4.50	0.730	−1.174	0.750		
	DV3	4.50	0.632	−0.904	0.875		
	DV4	4.75	0.447	−1.278	1.0		
Interest	INT1	4.75	0.577	−2.375	0.875		
	INT2	4.31	0.946	−1.266	0.625		
	INT3	4.19	1.109	−1.089	0.50		
	INT4	4.81	0.403	−1.772	1.0		
Efficiency	EFC1	4.31	0.873	−1.397	0.750		
	EFC2	4.56	0.727	−1.433	0.750		
	EFC3	3.94	1.289	−1.149	0.50		
	EFC4	4.00	0.894	−0.639	0.50		
	EFC5	4.50	0.816	−1.260	0.625		
Complexity	CMP1	4.06	1.063	−0.519	0.250		
	CMP2	4.38	0.806	−1.717	0.875		
	CMP3	4.63	0.619	−1.505	0.875		
Stability	STB1	4.81	0.403	−1.772	1.0		
	STB2	4.75	0.577	−2.375	0.875		
Relationship	RLS1	4.50	0.816	−1.260	0.625		
	RLS2	4.56	0.727	−1.433	0.750		
	RLS3	4.63	0.500	−0.571	1.0		
Connectivity	CNT1	4.25	1.183	−1.656	0.50		
	CNT2	4.69	0.479	−0.895	1.0		
	CNT3	4.75	0.577	−2.375	0.875		
	CNT4	4.56	0.629	−1.183	0.875		

*** *p* < 0.001.

**Table 6:** Outdoor Playground Design Criteria of Fundamental Movement Skills and Perceptual-motor abilities.

Sort	Items *(Outdoor playgrounds should be designed* …)
Effectiveness	for children to develop their potential kinetic and perceptual-motor ability factors
	to improve young children’s fundamental movement skills, self-achievement, satisfaction, and holistic development through perceptual-motor skills using playground facilities
Innovation	to be able to change periodically by the participation of users
	to have various types of amusement facilities created to reflect the developmental characteristics of young children
Diversity	for each of the subjects held in the facilities to bring out perceptual abilities by inducing users’ cooperating skills
	to use surrounding materials from the environment in designs so that children could be exposed to sensory and visual experiences
	to have users capable of playing alone, playing cooperatively, and playing in parallel
	to have users capable of physical activity play, comprehensive play, autonomous play, etc.
Interest	as a space that allows for self-activities and the maintenance of interest
	to stimulate children’s curiosity in different aspects such as materials and color designs
	to arrange facility and materials to play which could provide users with opportunities of challenges and adventures
Efficiency	to constantly interact and harmonize with the surrounding natural environment
	in a proper arrangement of space (facilities, equipment, etc.)—without interference of children’s perceptual-motor development
	as a space wide enough for infants to run and play catch and hitting without any obstacles
Complexity	as a space where fundamental motor strength, perception and motor development could be enhanced through physical activities
	with a collaborative governance of experts in related fields
Stability	robustly and securely for young children to be able to play alone
	with elements that have sustainable copper, height, and intensity as well as permanence in upheaving climate change
Relationship	with information for users of procedures and guides on how parents can integrate with children’s play in the playground
	at a place where young children can geographically and environmentally access easily
Connectivity	to allow users to recognize the motor relationship of their body and utilizing their body movements freely
	to relate the purpose of the design and the concept of perceptual-motor abilities keenly
	to induce users of natural acquirement of motor skills and achievement of balanced motor development

## Data Availability

The data that support the findings of this study are available from the corresponding author, upon reasonable request.

## Appendix A

**Table A1:** Questions for the In-depth Interview.

	Items
Current Status of Outdoor Playgrounds	How do you view the current management and constitution of outdoor playgrounds in general?
	If you think that there needs to be changes to the general outdoor playground form and constitution, in which aspects do you think the playgrounds need modification?
Ways to Design Outdoor Playgrounds	There are many theories that emphasize the importance of early childhood development. Do you think playground designs should take children development into account? If so, why do you think so?
	For designing outdoor playgrounds from the perspective of developmental theories, which theory do you think has the closest relevance?
	What do you think exemplary outdoor playgrounds should have? State ideas regarding different aspects such as the facilities and environment, the objective and philosophy in the design, etc.
	Please introduce any exemplary designs of outdoor playgrounds in and out of Korea that you would like to share.
Improvements and Requirements	From the perspective of children’s development, do you think the current outdoor playground is designed well? If not, what do you think are the critical problems?
	On a regular basis as an expert in infant and children research, or in play infrastructure and play environment research, do have you any point of hope for outdoor playground designs? Please share if you do.

## Appendix B

**Table A2:** Items Confirmed from Delphi Round 2.

Sort		Items
Effectiveness	1	To design a space for the development of young children’s potential mobility and perceptual motor abilities such as climbing, jumping down, hanging
	2	To design to improve young children’s fundamental movement skills, self-achievement, satisfaction, and holistic development through using playground facilities
Innovation	3	To implement the concept of a “user-oriented playground” so that the playground could periodically change use to the participation of users
	4	To design a space where various types of amusement facilities are created to reflect the developmental characteristics of young children
Diversity	5	To design facilities based on subjects that could induce perceptual abilities so that various complex functions could be performed
	6	To use surrounding materials from the environment in designs so that children could be exposed to sensory and visual experiences
	7	To design a space where users could play alone, play cooperatively, and play in parallel
	8	To design a space of facility and area where comprehensive play (where children with diverse body features and senses can integrate), physical activity play (where children can be free of limits on various forces and speeds), and autonomous play (which could be voluntary used to recreate play environment) are possible
Interest	9	To design a space that allows for self-activities and maintenance of interest
	10	To stimulate children’s curiosity in different aspects such as materials and color designs
	11	To design space by arranging facilities and elements that could provide children with opportunities of challenges and adventures
Efficiency	12	To pursue overall harmony utilizing the nature environment surrounding the playground and to design the space to constantly interact with the surroundings
	13	To study and design a proper arrangement of space (facilities, equipment, etc.) without interference of children’s perceptual-motor abilities development
	14	To design a space wide enough for infants to run and play catch and hitting without any obstacles
Complexity	15	To design a space where fundamental motor strength, perception, and motor development could be enhanced through physical activities
	16	Throughout the process of designing, to build a collaborative governance of direct and indirect experts on outdoor playgrounds
Stability	17	To robustly and securely design for young children to be able to play alone
	18	To design the facilities with elements that have sustainable copper, height, and intensity as well as permanence in upheaving climate change
Relationship	19	To design the space where various activities (imitating activities) or relationships (leader and assistants) can be formed between users.
	20	To inform users of procedures and guides to how parents can integrate with children’s play in the playground (to provide a place where children and their parents make memories)
	21	To locate and design the space where young children can access easily (geographically and environmentally)
Connectivity	22	To design the place to applicable for having children to recognize the motor relationship of their body and to utilize their body movements freely
	23	To relate the purpose of the design and the concept of perceptual-motor abilities development keenly
	24	To induce young children to naturally acquire motor skills and achieve balanced motor development
